# Automated Live-Cell Imaging of Synapses in Rat and Human Neuronal Cultures

**DOI:** 10.3389/fncel.2019.00467

**Published:** 2019-10-17

**Authors:** Matthew V. Green, Thomas Pengo, Jonathan D. Raybuck, Tahmina Naqvi, Hannah M. McMullan, Jon E. Hawkinson, Ezequiel Marron Fernandez de Velasco, Brian S. Muntean, Kirill A. Martemyanov, Rachel Satterfield, Samuel M. Young, Stanley A. Thayer

**Affiliations:** ^1^Department of Pharmacology, University of Minnesota Medical School, Minneapolis, MN, United States; ^2^Informatics Institute, University of Minnesota, Minneapolis, MN, United States; ^3^Institute for Therapeutics Discovery and Development, University of Minnesota, Minneapolis, MN, United States; ^4^Department of Neuroscience, Scripps Research Institute, Jupiter, FL, United States; ^5^Max Planck Florida Institute for Neuroscience, Jupiter, FL, United States; ^6^Department of Anatomy and Cell Biology, Iowa Neuroscience Institute, University of Iowa, Iowa City, IA, United States; ^7^Department of Otolaryngology, University of Iowa, Iowa City, IA, United States

**Keywords:** synapse loss, synaptogenesis, automated microscopy, image processing, viral transduction, human iPSC

## Abstract

Synapse loss and dendritic damage correlate with cognitive decline in many neurodegenerative diseases, underlie neurodevelopmental disorders, and are associated with environmental and drug-induced CNS toxicities. However, screening assays designed to measure loss of synaptic connections between live cells are lacking. Here, we describe the design and validation of automated synaptic imaging assay (ASIA), an efficient approach to label, image, and analyze synapses between live neurons. Using viral transduction to express fluorescent proteins that label synapses and an automated computer-controlled microscope, we developed a method to identify agents that regulate synapse number. ASIA is compatible with both confocal and wide-field microscopy; wide-field image acquisition is faster but requires a deconvolution step in the analysis. Both types of images feed into batch processing analysis software that can be run on ImageJ, CellProfiler, and MetaMorph platforms. Primary analysis endpoints are the number of structural synapses and cell viability. Thus, overt cell death is differentiated from subtle changes in synapse density, an important distinction when studying neurodegenerative processes. In rat hippocampal cultures treated for 24 h with 100 μM 2-bromopalmitic acid (2-BP), a compound that prevents clustering of postsynaptic density 95 (PSD95), ASIA reliably detected loss of postsynaptic density 95-enhanced green fluorescent protein (PSD95-eGFP)-labeled synapses in the absence of cell death. In contrast, treatment with 100 μM glutamate produced synapse loss and significant cell death, determined from morphological changes in a binary image created from co-expressed mCherry. Treatment with 3 mM lithium for 24 h significantly increased the number of fluorescent puncta, showing that ASIA also detects synaptogenesis. Proof of concept studies show that cell-specific promoters enable the selective study of inhibitory or principal neurons and that alternative reporter constructs enable quantification of GABAergic or glutamatergic synapses. ASIA can also be used to study synapse loss between human induced pluripotent stem cell (iPSC)-derived cortical neurons. Significant synapse loss in the absence of cell death was detected in the iPSC-derived neuronal cultures treated with either 100 μM 2-BP or 100 μM glutamate for 24 h, while 300 μM glutamate produced synapse loss and cell death. ASIA shows promise for identifying agents that evoke synaptic toxicities and screening for compounds that prevent or reverse synapse loss.

## Introduction

Synaptic connectivity changes in response to physiological stimuli, under neurodegenerative conditions, during development, and following exposure to drugs and toxins. Synapse loss correlates with cognitive decline in many neurodegenerative disorders, including Alzheimer’s disease and HIV associated neurocognitive disorder (Koffie et al., 2011; Saylor et al., 2016), and defective synaptogenesis underlies neurodevelopmental disorders such as autism (Guang et al., 2018). Decreases in synaptic spine density are implicated in the CNS toxicity produced by neurotoxins and the adverse effects of some medications (Miller et al., 2012; Nishijima et al., 2014; Huang and Song, 2019). Conversely, the synaptogenesis produced by drugs that stabilize mood and enhance cognition are associated with functional improvement (Kim and Thayer, 2009; Liu et al., 2012; Luine, 2016). Thus, an efficient approach to quantify synaptic connectivity will prove useful for studying the mechanisms of synaptopathies and screening for therapeutic and adverse drug effects.

Primary neurons in culture form physiological synaptic networks, providing an experimentally accessible system for studying synaptic function. Cells are viable for weeks in culture allowing synaptogenesis and synaptodendritic damage to be studied over time. Precise concentrations of drugs and toxins can be tested by additions to the cell growth media. High throughput production of primary neuronal cultures was recently described (Spicer et al., 2018). Approaches to quantify synaptic connections have focused on immunocytochemistry and the use of fluorescent reporters (Nieland et al., 2014). Immuno-labeling of synaptic proteins is scalable (Sharma et al., 2013; Nieland et al., 2014) but requires fixation, limiting sampling to a single point in time, precluding a repeated measures experimental design. A variety of genetically encoded synaptic reporter constructs are available to label specific structures with high signal to noise in live cells, enabling non-invasive, longitudinal synaptic imaging.

Neurons expressing enhanced green fluorescent protein (eGFP)-tagged synaptic proteins such as postsynaptic density 95-enhanced green fluorescent protein (PSD95-eGFP) and gephyrin-eGFP display fluorescent puncta that represent functional synaptic connections, enabling changes in the density of excitatory and inhibitory synapses to be tracked over time (Waataja et al., 2008; Hargus and Thayer, 2013; Fortin et al., 2014). Neurons expressing synaptic reporters exhibit significant changes in synaptic density following exposure to excitotoxins (Kim et al., 2008b; Waataja et al., 2008), inflammatory cytokines (Kim et al., 2011; Mishra et al., 2012), or peptide neurotoxins (Hargus and Thayer, 2013; Zhang et al., 2019) and these changes can be prevented or reversed pharmacologically (Shin et al., 2012). Synaptic changes detected *in vitro* predict functional outcomes *in vivo*. For example, a toxin that induced loss of excitatory synapses between rat hippocampal neurons in culture produced comparable decreases in spine density *in vivo* that was accompanied by impaired cognitive function (Raybuck et al., 2017). The utility of synaptic imaging for assessing network integrity and the ability of live-cell imaging to predict functional outcomes *in vivo* prompted us to automate a synaptic imaging assay using an approach applicable to laboratories interested in mechanistic studies, drug screening, and toxicity testing on synapses.

Automated synaptic imaging assay (ASIA) is an efficient approach to label, image, and analyze synapses between live neurons. It detects both changes in synapse number and cellular toxicity in live neurons. The system is built on a commercially available research microscope and analysis conducted with open source software. ASIA uses viral transduction of neurons cultured in 96 well plates to express fluorescent reporters, enabling simultaneous quantification of synapse density and cell viability. Neurons are imaged with an automated image acquisition protocol using either confocal or wide-field microscopy. Resulting images feed into a fully-automated batch-processing image-analysis protocol. ASIA was validated for quantifying synapse loss in the absence and presence of overt cell death. We highlight the potential use of ASIA for studying neuronal subtypes within a mixed culture and demonstrate its utility in cultures of human induced pluripotent stem cell (iPSC)-derived cortical neurons.

## Materials and Methods

### Materials

Materials were obtained from the following sources: Glutamax (catalog number: 35050061), Neurobasal-A medium (catalog number: 12349015), Propidium iodide (PI; catalog number: P3566), Dulbecco’s modified Eagle’s medium (DMEM; catalog number: 31053), Hanks’ balanced salt solution (catalog number: 14175), fetal bovine serum (catalog number: 26140), horse serum (catalog number: 16050), and penicillin/streptomycin (catalog number: 15140) were from Thermo Fisher Scientific (Carlsbad, CA, USA); N21 Max Media supplement (catalog number: AR008) from R & D systems; glass bottom 96 well plates (item no 655892) were from Greiner Bio One (Monroe, NC, USA); human recombinant brain-derived neurotrophic factor (hBDNF; catalog number: 78005), human recombinant glial cell-derived neurotrophic factor (hGDNF; catalog number: 78058), BrainPhys Neuronal Medium and SM1 (catalog number: 05792) from Stem Cell Technologies (Vancouver, BC, Canada); 2-bromohexadecanoic acid (2-BP; catalog number: 238422), L-glutamic acid monosodium salt hydrate (catalog number: G1626), lithium chloride (catalog number: L8895), Cytosine β-D-arabinofuranoside hydrochloride (AraC; catalog number: C6645) were from Millipore Sigma (St. Louis, MO, USA).

### Rat Hippocampal Culture

All animal care and experimental procedures were performed following the Guide for the Care and Use of Laboratory Animals published by the U.S. National Institutes of Health. Ethical approval was granted by the Institutional Animal Care and Use Committee of the University of Minnesota (protocol 1612-34372A). Hippocampal cultures were prepared from embryonic day 17 Sprague–Dawley rats (Charles River, Wilmington, MA, USA) as described previously (Zhang et al., 2019) with modifications to allow for optimal growth in 96 well plates. Timed pregnant rats were euthanized by CO_2_ inhalation with an institutionally approved and calibrated CO_2_ chamber. Pups from both sexes were removed and rapidly decapitated with sharp scissors. Hippocampi were removed and immediately placed in cold Ca^2+^ and Mg^2+^-free HEPES-buffered Hanks’ balanced salt solution. Tissue was then trypsinized in 3 ml TrypLE Express for 10 min at 37°C. Trypsin-containing buffer was then replaced with DMEM without glutamine, supplemented with 10% fetal bovine serum and penicillin/streptomycin (100 U ml^−1^ and 100 mg ml^−1^, respectively), and triturated using flame narrowed Pasteur pipettes to dissociate the cells. Cell suspension was then plated in glass bottom Greiner 96-well plates pre-coated with Matrigel (50 μl, 0.2 mg ml^−1^, BD Biosciences, Billerica, MA, USA). Cells were grown in a humidified atmosphere at 5% CO_2_ and 95% O_2_, pH 7.4 at 37°C. On day *in vitro* (DIV) 1, 75% of media was exchanged with DMEM supplemented with 10% horse serum and penicillin/streptomycin. Cultures were treated with 1 μM AraC on DIV 4 to suppress glial overgrowth. On DIV 8, 75% of media was exchanged with Neurobasal-A medium supplemented with 1% Glutamax and 2% N-21 Max. All cultures were maintained for at least 14 DIV before experiments.

### Human iPSC-Derived Cortical-Astrocyte Co-cultures

Ninty-six well plates containing human iPSC-derived cortical neurons were obtained from StemoniX (catalog #: BCARX-AA-0096; Maple Grove, MN, USA). Cells were received 8–15 weeks after differentiation. Seventy-five percent of transfer media was exchanged twice with BrainPhys neuronal medium supplemented with 1% SM1 and hBDNF and hGDNF (both at 20 ng ml^−1^). Media was replaced 75% every 2–3 days for 1–2 weeks to allow for transport recovery.

### Viruses

Virus mediated gene transfer was used to express reporter constructs in neurons growing in 96-well plates. A dual expression helper-dependent adenovirus (HdAd) was constructed to express PSD95, a scaffolding protein localized to the postsynaptic density of excitatory synapses, fused to enhanced green fluorescent protein (PSD95-eGFP) under the control of the human synapsin promoter (hSyn) and a separate neurospecific mCherry expression cassette also driven by hSyn. First, PSD95-eGFP was subcloned into the synapsin expression cassette (Lubbert et al., 2017). Briefly, this cassette includes the 470 bp hSyn, the mvm intron, and the BGH polyA. PSD95-eGFP was amplified using PfuUltra II Fusion high-fidelity DNA Polymerase (Agilent) from pGW1-CMV-PSD95-eGFP with primers containing 5^′^-EcoRI and 3^′^ NotI restriction enzyme sites (Forward: 5^′^-AGCTAGAATTCGCCACCATGGACTGTCTCTGTAT-3^′^, Reverse: 5^′^-TAGCTGCGGCCGCTTACTTGTACAGCTCGTCCATGCC-3^′^). The PSD95-eGFP insert and the synapsin expression cassette were subject to a double digest with EcoRI-HF and NotI-HF (New England Biolabs) followed by ligation with T4 DNA Ligase (New England Biolabs). Next, the two AscI restriction sites present in the PSD95 insert were subject to site-directed mutagenesis (QuikChange II Kit, Agilent) while maintaining the codons required for wild-type PSD95 expression (Forward 1: 5^′^-GTATAGTGACAACCCGGCGAGCCGAGCAAAAGCTGATATC-3^′^, Reverse 1: 5^′^-GATATCAGCTTTTGCTCGGCTCGCCGGGTTGTCACTATAC-3^′^, Forward 2: 5^′^-GGACTTGGGGCGAGCCAAGAAATACC-3^′^, Reverse 2: 5^′^-GGTATTTCTTGGCTCGCCCCAAGTCC-3^′^). Subsequently, the hSyn PSD95-eGFP expression cassette was cloned into the AscI site of pdelta 28E4 (Palmer and Ng, 2003), gift from Dr. Phil Ng, using In Fusion (Clontech). This version of pdelta28E4 contains a separate neurospecific mCherry expression cassette that is driven by the 470 bp hSyn promoter (Lubbert et al., 2017). The final HdAd plasmid allows for the expression of PSD95-eGFP independently of mCherry as a dual expression recombinant Ad vector. Production of HdAd was carried out as previously described (Montesinos et al., 2016). Briefly, pHdAd was linearized by PmeI and then transfected into 116 producer cells (Profection^®^ Mammalian Transfection System, Promega). For HdAd production, helper virus (HV) was added the following day. Forty-eight hours post-infection, after cytopathic effect (CPE), cells were subjected to three freeze/thaw cycles. Lysate was amplified in a total of five serial coinfections of HdAd and HV from 3 × 6 mm tissue culture dishes followed by a 15 cm dish and finally 30 × 15 cm dishes of 116 cells (confluence ~90%). HdAd was purified by CsCl ultracentrifugation and stored at −80°C in storage buffer (in mM): 10 HEPES, 250 sucrose, 1 MgCl_2_ at pH 7.4. Rat hippocampal neurons were infected with HdAd-hSyn-PSD95-eGFP-hSyn-mCherry at a final titer of 3.73 × 10^7^ virus particles (VP)/ml on DIV 8 and imaged on DIV 14–15. Human iPSC-derived neuronal cultures were infected with the HdAd at a final titer of 3.73 × 10^8^ VP/ml and imaged 6 days post infection.

Adeno-associated viruses (AAV) were produced by the Viral Vector and Cloning Core facility at the University of Minnesota following standard packaging procedures (Chen et al., 2019). pRC packaging plasmids for AAV1, 2, 5, 6, 9 and rh10 were obtained from the University of Pennsylvania Vector Core. The pRC-DJ packaging plasmid was obtained from Cell Biolabs (Cell Biolabs, Inc., San Diego, CA, USA). The pRC-PHP.eB packaging plasmid was a gift from Dr. Viviana Gradinaru (California Institute of Technology; Chan et al., 2017). PSD95.FingR-eGFP and GPHN.FingR-eGFP plasmids were a gift from Dr. Don Arnold (University of Southern California; Gross et al., 2013). The plasmid pAAV-CaMKIIα-hChR2(H134R)-EYFP (a gift from Dr. Karl Deisseroth—Addgene plasmid #26969) was used as the backbone for the cloning of the PSD95.FingR construct to allow selective expression in principal cells (Lee et al., 2010). The hSyn-eGFP plasmid was a gift from Dr. Bryan Roth (Addgene plasmid #50465). The pAAV-mDlx-mCherry plasmid was a modification of plasmid pAAV-mDlx-GCaMP6f (a gift from Dr. Gordon Fishell—Addgene plasmid #83899; Dimidschstein et al., 2016).

Cultures were infected with AAVs at the following titers: AAV1-hSyn-eGFP at 5.2 × 10^11^ genome copies (GC)/ml; AAV2-Syn-eGFP at 4.86 × 10^11^ GC/ml; AAV5-hSyn-eGFP at 3.95 × 10^12^ GC/ml; AAV6-hSyn-eGFP at 1.33 × 10^11^ GC/ml; AAV9-hSyn-eGFP at 2.07 × 10^12^ GC/ml; AAVDJ-hSyn-eGFP at 6.35 × 10^11^ GC/ml; AAVrh10-hSyn-eGFP at 3.48 × 10^11^ GC/ml; AAVPHP.eB-hSyn-eGFP at 2.27 × 10^12^ GC/ml; AAVPHP.eB-mDlx-mCherry at 3.06 × 10^11^ GC/ml; AAVPHP.eB-hSyn-PSD95.FingR-eGFP/mCherry at 2.82 × 10^11^ GC/ml, AAV8-CaMKIIα-FingR.PSD95-eGFP at 1.02 × 10^11^ GC/ml; and AAVPHP.eB-CAG-GPHN.FingR-eGFP at 3.64 × 10^11^ GC/ml. Rat hippocampal neurons were infected with AAVs on DIV 8 and imaged on DIV 21–22. Human iPSC-derived neuronal cultures were imaged 6 days post infection.

### Image Acquisition

**Figure 1:**
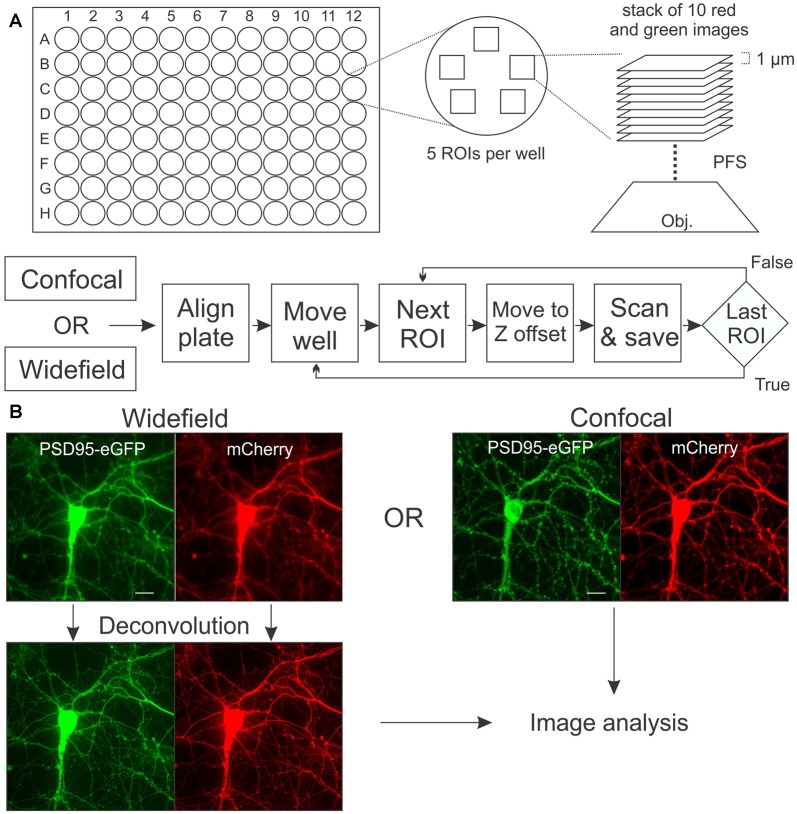
Image acquisition. **(A)** In each well of a 96-well plate, a stack of 10 images separated by 1 μM in the z-axis was collected from five regions of interests (ROIs). The perfect focus system (PFS) uses an IR laser to locate the bottom of the plate so that image acquisition begins at a precise z-offset from the bottom of the well. Flow chart shows sequence of alignment, movement and acquisition (scan) for automated microscope controlled by the JOBS module in Elements software. **(B)** Rat hippocampal cultures in 96-well plates were transduced using a bicistronic HdAd virus with a hSyn promoter driving independent expression of postsynaptic density 95-enhanced green fluorescent protein (PSD95-eGFP) (green) and mCherry (red). Images were collected with laser scanning confocal microscopy or with wide-field microscopy as described in “Materials and Methods” section. Maximum intensity projections (MIPs; shown) for the confocal and wide-field (after deconvolution) images are uploaded for subsequent image processing. Scale bar: 10 μM.

Images were acquired on a Nikon A1 confocal microscope (Nikon, Melville, NY, USA) using a 40 × (0.95 numerical aperture) air objective and controlled with the JOBS module of Nikon Elements software. eGFP was excited at 488 nm and emission detected at 550 nm (50 nm band pass). mCherry was excited at 561 nm and emission detected at 600 nm (50 nm band pass). For acquisition of wide-field images, the same microscope was fitted with a Photometrics Prime 95B CMOS camera and a Lumencor aura light engine LED light source. A penta-band pass filter set (Exciter: FF01-378/474/554/635/735-25; Emitter: FF01-432/515/595/681/809-25; Polychroic: FF-409/493/573/652/759; SemRock, Rochester, New York, NY, USA) was used to collect green and red fluorescence with wide-field microscopy. Representative images collected with wide-field and confocal microscopy are shown in Figure 1. Plates with cells were maintained at 37°C and 5% CO_2_ in a Chamlide stage-top incubator modified to hold 96-well plates that were mounted on a digitally controlled encoded stage. An acquisition protocol was built in JOBS and is shown in schematic form in Figure 1A. The protocol uses a plate alignment feature that was run each time a plate was placed on the stage. Images of the edge of three corner wells were acquired and used as fiducial markers to align the plate each time it was returned to the tissue culture incubator and reinstalled on the microscope, ensuring repeated imaging of precisely the same image fields. Prior to reading a plate for the first time an offset from the bottom of the plate to bottom of the cell layer was determined using an IR-laser-based z-positioning device to find the bottom of the plate. Thus, prior to starting each z-series the objective was positioned to focus on the bottom of the cell layer regardless of a tilt to the plate or warp in its glass bottom. For each region of interest (ROI) red and green image stacks were acquired, each composed of 10 frames spaced 1 μm apart in the z-axis. Multiple ROIs per well were collected, as detailed for each experiment. This design allows automatic acquisition of images from the same ROI over time. Using laser scanning confocal microscopy one well can be imaged (five ROIs per well, 10 steps per ROI) at a rate of about 4 min/well, whereas wide-field images can be collected at approximately 1 min/well (depending on exposure time).

### Image Analysis

Previously, we published an image analysis protocol to identify and count synapses using MetaMorph software (Molecular Devices, San Jose, CA, USA; Waataja et al., 2008). Here, we recreated the basic workflow in the open source software platforms, ImageJ and CellProfiler, and added analysis of a cell death marker to the workflow. Files for running analysis on all three platforms are provided in Supplementary Material, Datasheet 1 and can also be found with additional instructions for usage at https://github.com/thayerlab/ASIA-pipelines. All data presented in this article were analyzed with scripts written in ImageJ. The analyses in all three platforms were designed in a batch processing format to deal with the hundreds of ROIs imaged per plate. Additionally, images collected with wide-field microscopy require a deconvolution step before analysis, which was performed using a script written in ImageJ for batch processing (Supplementary Material, Datasheet 1). The deconvolution process was slow on a desktop computer utilizing ImageJ software, which does not utilize the computer’s GPU to accelerate processing. Thus, we processed the ImageJ script at the Minnesota Supercomputing Institute (MSI). Using the deconvolution script written in ImageJ with MSI processing, we were able to deconvolve 300 images in approximately 10 min, whereas this would have taken approximately 12.5 h on a single workstation (Xeon Silver 4110, 2.1 GHz). After deconvolution, images were further processed with ImageJ, CellProfiler, or MetaMorph.

**Figure 2:**
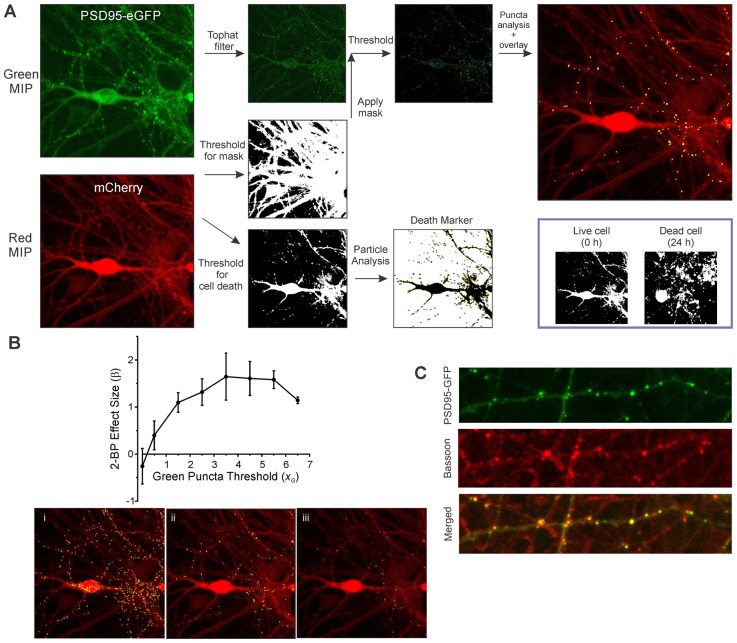
Image analysis workflow. **(A)** MIPs for the red and green channels were generated from ROIs containing neurons transduced with HdAd-hSyn-PSD95-eGFP-hSyn-mCherry. A threshold equal to 0.5 × image mean intensity was applied to the red image to create a binary mask. A top hat filter was applied to the green image to enhance contrast. The red mask and the filtered green image were combined *via* a logical AND function to remove green structures that were not in contact with the cell defined by the red channel. A threshold equal to the image mean intensity + 3.5 × image SD was applied to create an image containing structures representing synapses. Puncta counted as synapses (8–80 pixels) are shown overlaid on the mCherry MIP. Finally, a second threshold equal to the mean + 0.25 × image SD was applied to the red MIP and the resulting image used for particle analysis. Inset, a binary image of a cell before and after 24 h exposure to 300 μM glutamate is shown. Note that before treatment (0 h) much of the cell structure is intact and forms a contiguous structure. However, overt toxicity produces many small structures that result from the loss of membrane integrity as shown in the image collected at 24 h. **(B)** The optimal threshold setting for counting green puncta was determined from the SSMD [$\beta =(\mu_{1}-\mu_{2})/\surd (\sigma_{1}^{2}+\sigma_{2}^{2})$] for control (untreated) relative to 2-bromopalmitic acid (2-BP)-treated wells calculated from three separate 96-well plates. β is plotted vs. a scaling factor (*x*_G_) used to calculate the threshold which is defined as I_green_ + *x*_G_ (SD_green_) where I_green_ is the average intensity from the filtered green MIP and SD_green_ is the standard deviation from the filtered green MIP. Representative processed images show counted puncta (yellow) overlaid on mCherry MIP for threshold settings where *x*_G_ was equal to 1.5 **(i)**, 3.5 **(ii)**, and 5.5 **(iii)**. **(C)** Immunocytochemistry shows PSD95-GFP puncta co-localized with bassoon immunoreactive puncta. Neurons transduced with HdAd-hSyn-PSD95-eGFP-hSyn-mCherry were fixed and labeled with an antibody to bassoon as described in “Materials and Methods” section. Confocal images show PSD95-GFP (green channel), Bassoon immunoreactivity (far red channel) and merged image. Scale bar: 10 μM.

The sequential steps for image processing are described in Figure 2. Image analysis of both the green and red maximum intensity projection (MIP) images is fully batch processed. A morphological top hat filter set to the largest accepted puncta size is applied to the green MIP image to enhance puncta-like structures while decreasing the intensity of larger objects such as somata. A low threshold binary image derived from the red MIP (threshold was based on the mean intensity of the red MIP) was used as a mask *via* a logical AND function with the green MIP for puncta analysis. An intensity threshold (Otsu’s method) was then applied to the masked green image (threshold was based on the mean intensity and SD of the filtered green MIP) and the puncta meeting size criteria were counted. The maximum puncta size accepted was the same value used in the top hat filter. Particle analysis for assessing cell viability was performed on a high threshold binary image derived from the red MIP (threshold was based on the mean and SD of the intensity of the red MIP). As shown in Figure 2A (inset), structures in the binary image of live cells are mostly contiguous and after cell death induced by 300 μM glutamate the structures fragment.

Exact puncta size criteria and threshold values were dependent on software used (due to slight differences in the morphological top hat filter step as well as mask dilation and erosion steps) and type of reporter (eGFP fusion proteins tend to produce larger puncta than eGFP-tagged intrabodies). The threshold settings were optimized to detect the change in puncta number in untreated control wells relative to wells treated with a positive control stimulus. 2-bromopalmitate (2-BP) was used as a positive control stimulus for this first set of experiments. It prevents the synaptic targeting of PSD95 by inhibiting palmitoylation (El-Husseini et al., 2000a) and has previously been shown to reduce PSD95 puncta count (Waataja et al., 2008). To optimize effect size the strictly standardized mean difference (SSMD) was determined for untreated wells relative to 2-BP-treated wells on three separate 96-well plates. The SSMD (denoted as β) was defined as $\beta =(\mu_{1}-\mu_{2})/\surd (\sigma_{1}^{2}+\sigma_{2}^{2})$ where μ_1_ = mean change in puncta count from control wells, μ_2_ = mean change in puncta count from 2-BP-treated wells and $\sigma_{1}^{2}$ = the variance for the control change in puncta count and $\sigma_{2}^{2}$ = the variance for the 2-BP treated change in puncta count. As shown in Figure 2B, the optimal threshold for counting PSD95-eGFP puncta was equal to the mean intensity for the filtered green MIP + 3.5 (SD for the filtered green MIP). Processed images for a low threshold (Figure 2Bi), the optimal threshold (Figure 2Bii) and a high threshold (Figure 2Biii) are shown. The parameters used to analyze all the experiments in this study are presented in Supplementary Table S1.

### Immunocytochemistry

Rat hippocampal cultures were infected with HdAd-hSyn-PSD95-eGFP-hSyn-mCherry on DIV 8 as described above. On DIV 14, cells were washed with PBS and then fixed with methanol at −20°C for 10 min. Cells were then washed with PBS three times. After washing, cells were incubated with mouse anti-Bassoon monoclonal antibody (1:200, Enzo Life Sciences, Farmingdale, NY, USA) in 3% BSA blocking buffer at 4°C overnight. Cells were then washed three times with PBS and incubated with Alexa 647-conjugated goat anti-mouse antibody (1:500; Millipore, Billerica, MA, USA) in blocking solution at room temperature for 1 h. Cells were imaged after two washes. PSD95-GFP was excited at 488 nm and emission was collected from 500 to 550 nm. Alexa 647 was excited at 640 nm and emission was collected from 650 to 720 nm. Co-localization analysis of GFP and Alexa 647 puncta was performed as follows. Each image stack was deconvolved using SVI Huygens Pro (CmlE, theoretical PSF, default parameters) before downstream analysis. The PSD95-GFP and Bassoon channels were filtered using a Laplacian of Gaussian with a sigma of 0.15 μM in X and Y, 0.45 μM in Z. A threshold of half the maximum intensity after filtering was used to identify regions of local maxima. Each connected component after thresholding was considered a separate punctum. For each punctum in the PSD95-GFP channel, we analyzed for the presence of a punctum in the bassoon channel by measuring the amount of overlap with the region above threshold. An overlap of 10% was considered apositive co-occurrence.

### Statistics

All statistics were performed using RStudio (Boston, MA, USA) and Prism, GraphPad 8 software (La Jolla, CA, USA). Data distributions were first tested using the Kolmogorov–Smirnov test for normality and Bartlett’s test for homogeneity of variance. For two group comparisons, a nested unpaired *t*-test was used with each image ROI defined as an individual sample (*n* = 1), nested within wells. If data were normal and variance considered equal, and if data contained more than two groups, nested analysis of variances (ANOVAs) were used with each image ROI defined as an individual sample (*n* = 1), nested within wells. If variances were considered unequal or data was not normal, data were transformed if necessary using a log transformation and a Kruskal–Wallis test was used to determine differences. Time-course data were first analyzed with a multi-way repeated measures ANOVA followed by a one-way ANOVA to compare data collected at each time point. The number of samples (N) are presented in the figure legends.

## Results

### Validation of the Assay

We have previously published results indicating that PSD95-GFP puncta represent functional excitatory synapses. The PSD95-GFP puncta co-localize with functional presynaptic sites for neurotransmitter release and they co-localize with synaptically driven postsynaptic Ca^2+^ increases (Waataja et al., 2008). PSD95-GFP puncta co-localize with N-Methyl-D-aspartate (NMDA) receptor immunoreactivity and after treatments that increase the number of puncta, the new puncta also co-localize with NMDA receptors (Kim and Thayer, 2009). A similar live-cell assay that measured the presynaptic marker synaptophysin fused to eGFP detected loss of presynaptic terminals in response to the same stimuli that evoked loss of PSD95-GFP (Shin and Thayer, 2013). NMDA-induced loss of PSD95-GFP puncta correlates with loss of evoked EPSC amplitude and drugs that protect the number of puncta also prevent the decrease in EPSC amplitude (Waataja et al., 2008). In Figure 2C, we show images of rat hippocampal cultures transduced with HdAd-hSyn-PSD95-eGFP-hSyn-mCherry, then fixed and labeled with an antibody to the presynaptic protein bassoon. Analysis of these immunocytochemistry results indicated that 76 ± 8% (*n* = 5) of PSD95-GFP puncta overlapped by at least 10% with a bassoon immunoreactive punctum. We interpret this result to indicate that the majority of PSD95-GFP puncta represent synapses with presynaptic elements and note that the analysis likely underestimates the total number of synaptic pairs because our co-localization criteria required 10% overlap.

**Figure 3:**
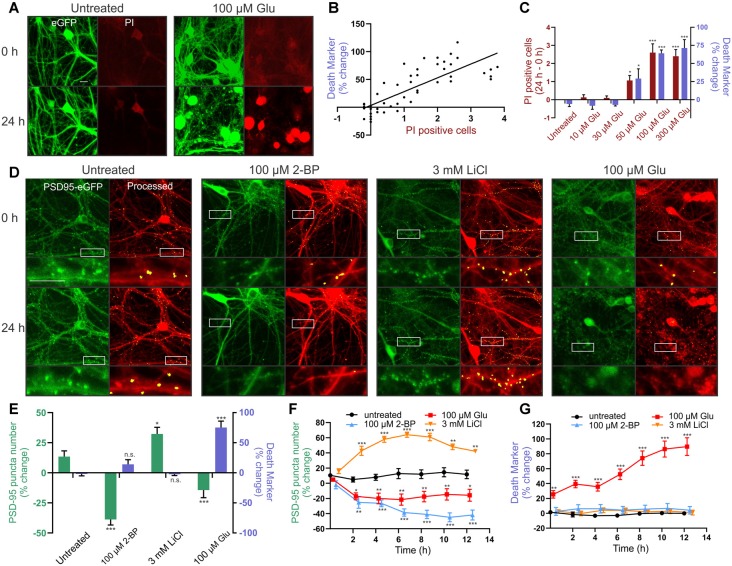
Reliable detection of synaptic changes and cellular toxicity in rat hippocampal cultures. **(A–C)** Cultures were transduced with AAVPHP.eB-hSyn-eGFP and imaged in the presence of 5 μg/ml propidium iodide (PI). **(A)** Representative images show eGFP (green) and PI (red) fluorescence before (0 h) and after (24 h) no treatment (untreated) or treatment with 100 μM glutamate (Glu). Scale bar: 10 μM. **(B)** The death marker (particle number) determined from the eGFP binary image correlated with the number of PI-positive cells in the same field (Linear regression; *r*_(46)_ = 0.6351, *p* ≤ 0.0001, *n* = 36 wells). **(C)** Glutamate elicited a concentration-dependent increase in the death marker (particle number; purple bars) and number of PI-positive cells (red bars) over the course of 24 h (Kruskal–Wallis test; K–W statistic = 25.68, *p* ≤ 0.0001 for particle number. Kruskal–Wallis test; K–W statistic = 27.36, *p* ≤ 0.0001 for number of PI-positive cells, *n* = 6 wells per group). **(D,E)** Cultures were transduced with HdAd-hSyn-PSD95-eGFP-hSyn-mCherry.** (D)** Representative images show PSD95-eGFP (green) and the processed overlay (puncta in yellow, mCherry MIP in red) for ROIs before (0 h) and 24 h following no treatment (untreated) or treatment with 100 μM 2-BP, 3 mM LiCl, or 100 μM glutamate. Insets display enlarged images of the boxed regions. Scale bar: 10 μM. **(E)** Bar graph displays change in synapse number (PSD95 puncta) and change in death marker (particle number) 24 h after treatment under the indicated condition (nested one way analysis of variance, ANOVA; *F*_(3,32)_ = 17.48, *p* ≤ 0.0001 for particle number, nested one way ANOVA; *F*_(3,32)_ = 31.84, *p* ≤ 0.0001 for puncta change, *n* = 42 ROIs from nine wells for untreated, *n* = 41 ROIs from nine wells for 3 mM LiCl, *n* = 43 ROIs from nine wells for 100 μM 2BP, *n* = 40 ROIs from 9 wells for 100 μM glutamate). **(F,G)** Time-lapse experiments show change in synapse number (PSD95 puncta) and death marker (red mask particle number) compared to initial baseline images during an automated repeated acquisition experiment. Image values as a percent of baseline are plotted vs. time post-treatment (one-way ANOVA followed by Tukey’s *post hoc* test; *p < 0.05, **p < 0.01, ***p < 0.001 compared to untreated, *n* = 9 wells per group with five ROIs averaged per well). Data were collected with laser scanning confocal microscopy. ImageJ settings were as follows; Red MIP mask threshold: 0.25 × red MIP mean intensity, Red MIP threshold for particle number: Red MIP mean + 0.25 × SD, Puncta threshold: green MIP mean + 3.5 × SD, puncta size restriction: 8–80 pixels. Data are expressed either as mean counts from individual wells **(B)** or as mean ± standard error of the mean (SEM) of each ROI **(C,E,F,G)**. Kruskal–Wallis tests were followed by Dunn’s *post hoc* test, one-way ANOVAs were followed by Tukey’s *post hoc* test, n.s. = not significant, *p < 0.05, **p < 0.01, ***p < 0.001 compared to untreated.

We tested the reliability of ROI particle number in the binary image derived from the red MIP as a marker for cell death. PI labels the nucleus of cells that have lost membrane integrity and is a widely used marker of cell death (Lau et al., 2007). Because PI fluorescence overlaps with that of mCherry, we made an AAV serotype PHP.eB (AAV-PHP.eB) that expressed eGFP behind the hSyn promoter. Transduced cells (Figure 3A, green) were used to generate a binary image for counting particles, similar to how the mCherry MIP was processed in Figure 2 (inset). PI was imaged in the red channel from the same ROIs. We then treated the cells for 24 h with increasing concentrations of glutamate to induce neuronal death and determined whether the number of PI-positive cells correlated with the number of particles counted in the binary image. The change in the number of particles counted in GFP binary images acquired before and 24 h after glutamate treatment correlated with the change in the number of PI-positive cells imaged in the same ROI (Figure 3B). Note that the percent change in the death marker is a change in the number of particles not explicitly a percent change in survival. These values are correlated but not identical. Cell death was not detected with either PI or particle analysis in untreated wells, or wells treated with 10 μM or 30 μM glutamate, but significant increases in PI and increases in particle number appeared in ROIs treated with 50 μM glutamate and peaked at 100 μM glutamate (Figure 3C).

Next, we tested whether the assay would reliably detect changes in the number of synapses in rat hippocampal cultures under a variety of conditions. We treated cultures with 2-bromopalmitic acid (2-BP) to decrease synapse number, with lithium to increase synapses, and with glutamate to decrease synapse number coincident with death. First, we used 2-BP to evoke synapse loss in the absence of cell death. Untreated wells displayed a slight increase in puncta count (13%) over 24 h. Treatment with 100 μM 2-BP for 24 h reduced puncta number by 52 ± 5% as determined by the automated assay (Figures 3D,E). The significant loss of synapses produced by 2-BP occurred in the absence of any change in particle number, the marker for cell death. To show that the assay can be used to study synaptogenesis we treated neurons with 3 mM lithium which has previously been shown to increase excitatory synapse number (Kim and Thayer, 2009). Twenty-four hours treatment with 3 mM LiCl increased the number of puncta by 19 ± 6%, a significant increase compared to untreated wells. LiCl did not affect the cell death marker (Figures 3D,E). Finally, to address the confounding phenomenon of cell death in the assay, and the ability of the particle count to detect dying cells, we used glutamate treatment to provide an example of synapse loss occurring concurrently with cell death. Treating rat hippocampal neurons with 100 μM glutamate caused a 14 ± 6% decrease in puncta number, a significant loss compared to untreated wells, but also elicited a 77 ± 10% increase in particle number in the mCherry binary image, indicating significant cell death (Figures 3D,E). Thus, the assay reliably detects changes in synapse number and can detect cell death when it accompanies synapse loss, an important feature when studying synaptic changes associated with neurodegenerative or toxic conditions. These treatments evoked changes in synapse number without significantly affecting the size or intensity of the counted puncta.

The automated system can repeatedly image the same ROIs allowing acquisition of robust time-course data sets. To demonstrate this capability, we acquired baseline images to determine initial puncta counts, applied treatments to the plate, and then started automated image acquisition. Subsequent images were collected every 2 h over the course of 14 h. Synaptic changes occurred largely within the first 4–8 h, as seen in Figure 3F. The number of synapses in ROIs from untreated wells was stable (Figure 3F). Particle analysis (Figure 3G) showed that the overt toxicity evoked by 100 μM glutamate exhibited an initial rapid component that began prior to acquisition of the first image after treatment (approximately 15 min) followed by a graded increase over the following 10 h. Survival in control (untreated) ROIs and those treated with 2-BP or LiCl was stable over the 14 h experiment. This highlights the potential for both long-term repeated imaging sessions to detect graded changes in synaptic connections under neurodegenerative or synaptogenic conditions as well as the ability to sample rapidly at shorter time scales using automated imaging.

### Other Uses for ASIA

**Figure 4:**
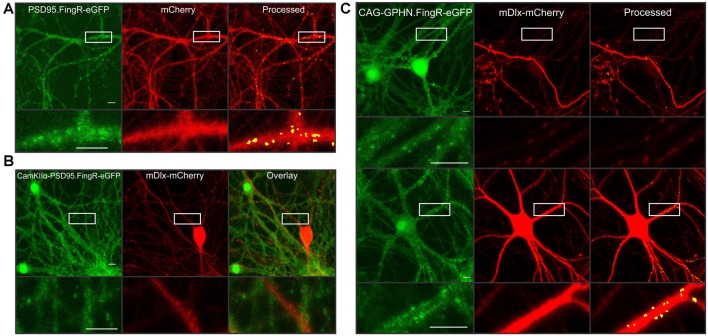
Highlighting the flexibility of automated synaptic imaging assay (ASIA). **(A)** Sample images show FingR.PSD95-eGFP (green), mCherry (red), and counted puncta (yellow) overlaid on mCherry MIP (processed). Images are representative of >100 image sets. Scale bar: 10 μM. ImageJ settings were as follows; Red MIP mask threshold: 0.25 × red MIP mean intensity, Red MIP threshold for particle number: Red MIP mean + 0.25 × SD, Puncta threshold: green MIP mean + 2.5 × SD, puncta size restriction: 4–60 pixels. **(B)** Sample images show restrictive expression with no overlap between excitatory neurons labeled with CamKIIα-FingR.PSD95-eGFP (green) and inhibitory neurons labeled with mDlx-mCherry (red) and overlay. Images are representative of four image sets. Scale bar: 50 μM. **(C)** Sample images show two principal neurons (upper panel) and an inhibitory neuron (lower panel) expressing CAG driven GPHN.FingR-eGFP (green). mDlx driven mCherry (red) is excluded from expression in presumed principal neurons (upper panel) but shows high-intensity expression in presumed inhibitory neuron (lower panel). Using identical threshold values, the red mask successfully limits puncta counted to those in the mDlx-mCherry positive neuron (Processed, lower panel) while excluding puncta expressed in the presumed principal neurons (upper panel). Insets display enlarged images of the boxed regions. An mDlx-mCherry positive process that originates from a cell outside of the field traverses the ROI shown in upper panel (red). Images are representative of six image sets. Scale bar: 10 μM. ImageJ settings were as follows; Red MIP mask threshold: red MIP mean intensity + SD, Red MIP threshold for particle number: Red MIP mean + 0.25 × SD, Puncta threshold: green MIP mean + 2 × SD, puncta size restriction: 4–60 pixels.

A major advantage of the automated synapse counting assay is that it can be used with different reporters making it very flexible. Here, we highlight additional constructs generated in AAV vectors to selectively study various features of the neural network. PSD95 overexpression is known to induce the formation of new synapses (El-Husseini et al., 2000b). A fibronectin antibody-like protein labels endogenous PSD95 (PSD95.FingR-eGFP) in live cells without affecting PSD95 expression (Gross et al., 2013) and has previously been used by our lab and others to study synaptic changes (Lin et al., 2017; Zhang and Thayer, 2018). Thus, we packaged an AAV containing both PSD95.FingR-eGFP and mCherry driven by the hSyn promoter to study endogenous PSD95 clusters (Figure 4A). Minor changes to the puncta size and threshold level for the green image were needed in order to accurately detect intrabody puncta (see Figure 4 legend). Consistent with studies using the manual imaging assay (Zhang and Thayer, 2018), the automated assay accurately identified endogenous excitatory synapses.

The use of the binary mask derived from the red channel to restrict analysis of the green channel to puncta in contact with cells expressing the red fluorophore provides two advantages. First, by developing separate viruses for the red and green reporter constructs, a single red construct can be paired with multiple green reporter constructs. Second, because the assay only counts puncta that are within the red mask, the red construct can be driven by different cell-specific promoters to restrict the assay to counting puncta selectively in specific cell types. For example, we packaged AAVs containing either CaMKIIα-PSD95.FingR-eGFP or mDlx-mCherry to demonstrate restricted expression in excitatory neurons (Figure 4B, green) or inhibitory neurons (Figure 4B, red), respectively. Expression of PSD95.FingR-eGFP did not co-localize with mDlx-mCherry (Figure 4B, overlay) confirming the selectivity of these promoters.

To demonstrate the use of the binary mask to restrict counted synapses to a subset of neurons in the field, we packaged a virus containing the expression construct for a CAG-driven fibronectin intrabody targeted to gephyrin (GPHN.FingR-eGFP), a structural protein located at GABAergic synapses. In Figure 4C (green), images show GPHN.FingR-eGFP expression driven by the CAG promoter. The upper example shows two principal neuron somata expressing GPHN.FingR-eGFP. The lower panel shows an inhibitory neuron expressing GPHN.FingR-eGFP. When paired with cells transduced with mDlx driven mCherry the threshold for the red mask can be set to selectively count puncta from inhibitory neurons expressing mDlx-mCherry, whereas puncta from principal neurons not expressing mDlx-mCherry were excluded from analysis. Thus, numerous puncta are counted in the lower panel that contains an mDlx-mCherry positive neuron whereas in the upper panel only a few puncta are counted along a process crossing the ROI. These examples demonstrate the use of ASIA for studying various aspects of the synaptic content of neural networks. Using cell-specific promoters to drive the expression of the mask protein limits synapse counting to a subset of cells in the culture and the choice of reporter enables targeting of specific subcellular domains.

### Imaging Synapses Between Human iPSC-Derived Cortical Neurons

**Figure 5:**
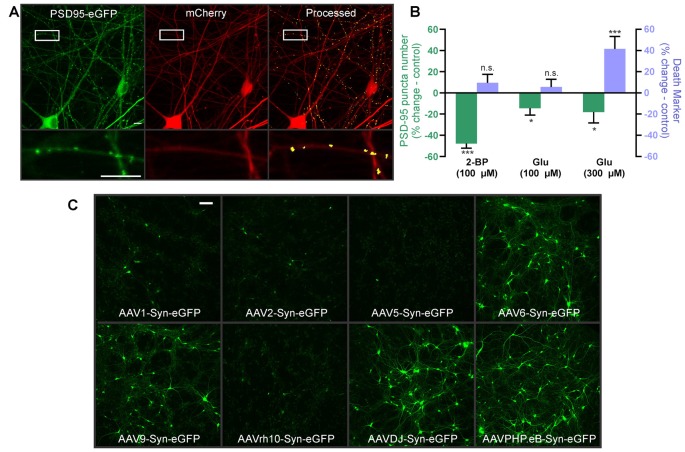
Automated synapse analysis in human induced pluripotent stem cell (iPSC)-derived neuronal cultures. **(A)** Representative confocal image of human iPSC-derived cortical neurons expressing HdAd-hSyn-PSD95-eGFP-hSyn-mCherry. Expression of the PSD95-eGFP fusion protein produces fluorescent puncta at excitatory synapses (green) and mCherry expression fills the cell and defines its morphology (red). Automated image processing was performed as described in Methods and puncta meeting size and intensity criteria were counted (yellow) and overlaid on the mCherry MIP (processed). Insets display enlarged images of the boxed regions. Scale bar: 10 μM. **(B)** Bar graph of change in synapse number (PSD95 puncta) and death marker (red mask particle number) from 0 to 24 h in neurons treated with 100 μM 2-BP, 100 μM glutamate, and 300 μM glutamate compared to respective untreated control wells. Data were collected using wide-field microscopy (nested unpaired *t*-test; *t*_(4)_ = 3.947, *p* = 0.017 for puncta change, *t*_(4)_ = 0.978, *p* = 0.383 for cell death, *n* = 36 ROIs from three wells for untreated, *n* = 35 ROIs from three wells for 100 μM 2-BP. Nested unpaired *t*-test; *t*_(10)_ = 2.291, *p* = 0.045 for puncta change, *t*_(10)_ = 0.849, *p* = 0.416 for cell death, *n* = 71 ROIs from six wells for untreated, *n* = 71 ROIs from six wells for 100 μM glutamate. Nested unpaired *t*-test; *t*_(10)_ = 2.277, *p* = 0.024 for puncta change, *t*_(10)_ = 2.214, *p* = 0.028 for cell death, *n* = 71 ROIs from six wells for untreated, *n* = 66 ROIs from six wells for 300 μM glutamate). ImageJ settings were as follows; Red MIP mask threshold: 0.5 × red MIP mean intensity, Red MIP threshold for particle number: Red MIP mean + 0.25 × SD, Puncta threshold: green MIP mean + 5 × SD, puncta size restriction: 3–30 pixels. Data are expressed as mean ± SEM of each ROI, n.s. = not significant, *p < 0.05, ***p < 0.001 compared to respective untreated control wells. **(C)** Images representative of three wells show hSyn-driven expression of eGFP in human iPSC-derived cortical neurons transduced with the indicated adeno-associated viruses (AAV) serotypes. Scale bar: 100 μM.

Human iPSC-derived neuronal cultures are becoming more widely available and effectively model neurodevelopmental and neurodegenerative synaptopathies (Taoufik et al., 2018; Shen et al., 2019). They show promise for drug and toxicity screening (Anson et al., 2011; Jorfi et al., 2018; Sirenko et al., 2019), but to date, live-cell synaptic imaging has not been used to screen for synaptic toxicity in human iPSC-derived neurons. We obtained human iPSC-derived cortical neuron-astrocyte co-cultures plated in 96 well plates from StemoniX. They form excitatory and inhibitory synapses and display spontaneous synaptically-driven activity (Sirenko et al., 2019). We infected the iPSC-derived neurons with HdAd-hSyn-PSD95-eGFP-hSyn-mCherry and observed expression of both mCherry and a punctate pattern of PSD95-eGFP that could be processed by the analysis software (Figure 5A). Next, we tested whether we could measure synapse loss in the iPSC-derived cortical neurons following exposure to 2-BP. Indeed, 24 h treatment with 100 μM 2-BP caused a 48 ± 5% decrease in puncta number compared to controls without significantly affecting cell viability. Thus, human iPSC-derived neurons form synapses that are sensitive to positive control treatments comparable to the rat neurons (Figures 3D,E). Next, we used a more physiologically relevant treatment to test whether the human neurons would exhibit excitotoxicity. The iPSC-derived cortical neurons responded to glutamate, although at higher concentrations than the rat neurons. Interestingly, we found that 100 μM glutamate decreased the number of synaptic puncta by 15 ± 5% compared to untreated wells without eliciting significant cell death in the human iPSC-derived neurons. In contrast, 300 μM glutamate caused both an 18 ± 7% decrease in puncta and a 42 ± 8% increase in mCherry particle number, indicating significant cell death in the human iPSC-derived neurons following treatment with the higher concentration of glutamate (Figure 5B). Thus, this assay can reliably distinguish changes in synapse number from overt cellular toxicity in human iPSC-derived neuronal cultures. This assay can be used to detect subtle changes in synapse density in human neurons that underlie neuropsychiatric disorders.

Finally, because AAV tropism in human iPSC-derived cortical neurons has not been thoroughly investigated and because different AAV serotypes optimally infect different species (Duong et al., 2019), we generated a panel of different AAV serotypes to determine the optimal vector for studying the human iPSC-derived neurons. Because preliminary experiments with HdAd-hSyn-PSD95-eGFP-hSyn-mCherry established that the hSyn promoter successfully expresses in human iPSC cultures, we packaged a series hSyn-eGFP expressing AAV serotypes. We tested eight common AAV serotypes: AAV1, AAV2, AAV5, AAV6, AAV9, AAVrh10, AAVDJ, and AAVPHP.eB. AAV 8 was not tested because it did not infect the human iPSCs in pilot experiments with AAV8-hSyn.FingR.PSD95/mCherry and AAV8-CAG-tdTomato. Final virus titers used for each virus are reported in the “Materials and Methods” section, and were similar. AAVs 6, 9, DJ, and PHP.eB expressed at high levels and seem well suited for use in the human iPSC-derived neurons while AAVs 1, 2, 5, and 10RH did not express well in the human neurons (Figure 5C). Thus, future constructs created with AAV backbones for use in human iPSCs should use one of the four serotypes described here.

## Discussion

ASIA acquires high magnification images repeatedly from the same ROIs with sufficient precision and resolution to identify and track individual synaptic connections over time in live neurons. Image processing and analysis of each ROI yields quantitative measures of changes in the number of synapses and cell viability. The system is built on a commercially available research microscope and open source software making it widely accessible. Validation experiments in primary cultures of rodent neurons and human iPSC-derived cortical neurons demonstrated quantitative assessment of synapse loss and synaptogenesis in the absence of changes in viability. Proof of concept studies highlight the use of cell-specific promoters to produce a binary mask to selectively study subsets of cells in the culture and reporters that target distinct subcellular domains were used to image inhibitory or excitatory synapses.

We validated key features of ASIA including its ability to detect changes to both synaptic density and cell viability. Treatment with 2-BP and lithium chloride significantly decrease and increase, respectively, excitatory synapse number without affecting cell survival. Synapse density can be highly variable *in vitro*; thus, having multiple time points from the same ROI improves statistical power in an otherwise variable assay. This approach also makes ASIA well-suited to performing time-course studies. Time-course experiments are particularly valuable during assay development, to establish cause and effect, and to measure transient events. ASIA can detect cell death automatically as exemplified by excitatory synapse loss and cellular toxicity evoked by glutamate. Interestingly, the human iPSC-derived neuronal cultures treated with 100 μM glutamate exhibited synapse loss in the absence of the cell death that was observed during exposure to higher glutamate concentrations (Figure 5B). Distinguishing the subtle changes in synaptic density expected in models of synaptopathies (Kim et al., 2008a) from synapse loss accompanied by overt cell death is particularly useful in mechanistic studies. In toxicity screens, this information might help separate compounds that evoke subtle behavioral phenotypes from those eliciting gross brain damage. Analyzing other features in the images will yield additional disease-relevant information. For example, analysis of changes in cell morphology, such as dendritic beading and cellular blebbing, could quantify early stages of necrosis and changes in nuclear structure are commonly extracted in high content analysis approaches to detect apoptosis (Anilkumar et al., 2017). Because the ImageJ and CellProfiler platforms are widely used for high content image analysis (Vokes and Carpenter, 2008; Smafield et al., 2015), the workflows described here may easily be expanded to analyze additional features.

The viral transduction approach used in ASIA provides flexibility on many levels. It is relatively inexpensive to generate new viruses facilitating experimentation. The PSD95-eGFP construct is an example of the use of a tagged fusion protein to quantify the number of excitatory synapses, and demonstrates the advantage of the large packaging capacity of HdAd, which permits the expression of both the mask protein and the synaptic marker in one vector. Alternatively, the AAV-GPHN.FingR-eGFP expresses a fibronectin intrabody that labels inhibitory synapses and requires a separate virus to express the mask. The fibronectin antibody-like protein does not alter the expression of gephyrin or affect its function (Gross et al., 2013), a clear advantage over fusion proteins for certain experiments studying the mechanism of synaptic changes. The use of a separate construct to express the mask protein allows the use of different cell-specific promoters to limit analysis to certain cell types, such as inhibitory neurons identified with the mDLx promoter in Figure 4C. We provide just a few examples of the many possible experimental designs that can be used with ASIA, which is not limited to labeling architectural scaffolding proteins. For example, the Phlourin-tagged AMPA receptor (AMPAR; pCI-SEP-GluR1; Kopec et al., 2006), which decreases fluorescence intensity upon internalization from the plasma membrane into acidic endosomes, may prove useful with ASIA. Simple changes to analysis outputs to include puncta intensity and size will facilitate the study of AMPAR-mediated phenomena associated with synaptic plasticity. Finally, viral transduction enables flexibility in the choice of neuronal culture. Rodent primary neuronal cultures are well characterized and known to develop physiological synapses. Viruses provide a straightforward method of gene transfer without the need to develop transgenic lines. We did not demonstrate their use here but the ASIA is clearly suitable to work with neuronal cultures derived from animal models of disease.

We also demonstrated the use of ASIA with human iPSC-derived neuronal cultures because not all properties of human synapses are replicated in rodents. Synaptic activity-driven transcription in human and mouse cultures exhibit unique gene expression profiles (Pruunsild et al., 2017) and human iPSC models of Alzheimer’s disease produce tau neurofibrillary tangles lacking in mouse models (Choi et al., 2014). Human iPSC-derived cells effectively model neurodevelopmental and neurodegenerative synaptopathies (Taoufik et al., 2018; Shen et al., 2019) and show promise for drug and toxicity screening (Anson et al., 2011; Jorfi et al., 2018; Sirenko et al., 2019). Here, we establish that iPSC-derived cortical neurons can be transduced to label excitatory synapses in live cells. Additionally, we establish that the human iPSCs exhibit synapse loss using 2-BP and a more physiologically relevant stimulus, glutamate. The same analysis workflow used for rat neurons was able to detect synapse loss and cell death due to glutamate in the human iPSC-derived neuronal culture. To take advantage of the additional reporter constructs and cell-specific promoters we tested a panel of AAV serotypes and found four that successfully infected the human iPSCs (AAVs 6, 9, DJ, and PHP.eB).

ASIA provides a powerful approach for studying the mechanisms of synapse loss and synaptogenesis as well as for performing limited screens for drugs and toxins that affect synaptic density. The approach can easily be scaled to high density plate formats with simple changes to well and ROI position in the image acquisition software. The ASIA employs a workstation approach that is not suitable for fully automated (hands free) high throughput screening but, provides an effective platform for pilot studies. In the experiments described here, 2-BP produced significant synapse loss with an effect size large enough to potentially design a screen with feasible sample sizes (*n* = 5 ROIs) and power (0.8) to achieve significance (*p* < 0.05). The current assay is robust (*Z’* = 0.23; data from Figure 5), although requires some optimization to achieve a *Z’* > 0.5, which is generally regarded as suitable for high throughput screening. However, treatments with lithium produced smaller effect size and would require a much larger sample size (*n* = 33 ROIs) to observe significance (*p* < 0.05) with a large enough power (0.8), and would not be suitable for a screening assay (*Z’* = −0.65; data from Figure 3). Thus, ASIA can be used to determine the suitability of specific treatments and conditions for potential use in screening compound libraries. ASIA is well suited to perform pilot screens to evaluate particular choices of cell type, reporter construct and treatment response for use in high throughput screening.

ASIA is an efficient and flexible system to study changes in synaptic connections. We validated its ability to quantify synapse loss, synaptogenesis, and cell viability. Repeated imaging of the same ROI enables repeated measures experimental design increasing statistical power. Here, we highlighted potential uses for the assay and anticipate that it will provide a foundation for future synaptic imaging approaches that take advantage of novel fluorescent reporters and the development of disease-based human iPSC models.

## Data Availability Statement

The datasets generated for this study are available on request to the corresponding author.

## Ethics Statement

All animal care and experimental procedures were performed following the Guide for the Care and Use of Laboratory Animals published by the U.S. National Institutes of Health. Ethical approval was granted by the Institutional Animal Care and Use Committee of the University of Minnesota (protocol 1612-34372A).

## Author Contributions

MG and ST designed the experiments. MG, HM and TN performed the experiments. MG, ST and JH analyzed the experiments. MG, TP and JR developed the software. EM, KM, BM, RS and SY designed and prepared viral vectors. MG and ST wrote the manuscript.

## Conflict of Interest

The authors declare that the research was conducted in the absence of any commercial or financial relationships that could be construed as a potential conflict of interest.

## References

[B1] Anilkumar U. Weisova P. Schmid J. Bernas T. Huber H. J. Düssmann H.. (2017). Defining external factors that determine neuronal survival, apoptosis and necrosis during excitotoxic injury using a high content screening imaging platform. PLoS One 12:e0188343. 10.1371/journal.pone.0188343 29145487 PMC5690623

[B2] Anson B. D. Kolaja K. L. Kamp T. J. (2011). Opportunities for use of human iPS cells in predictive toxicology. Clin. Pharmacol. Ther. 89, 754–758. 10.1038/clpt.2011.9 21430658 PMC3593635

[B3] Chan K. Y. Jang M. J. Yoo B. B. Greenbaum A. Ravi N. Wu W. L.. (2017). Engineered AAVs for efficient noninvasive gene delivery to the central and peripheral nervous systems. Nat. Neurosci. 20, 1172–1179. 10.1038/nn.4593 28671695 PMC5529245

[B4] Chen S. H. Haam J. Walker M. Scappini E. Naughton J. Martin N. P. (2019). Production of viral constructs for neuroanatomy, calcium imaging and optogenetics. Curr. Protoc. Neurosci. 87:e66. 10.1002/cpns.66 30883041 PMC6530799

[B5] Choi S. H. Kim Y. H. Hebisch M. Sliwinski C. Lee S. D’Avanzo C.. (2014). A three-dimensional human neural cell culture model of Alzheimer’s disease. Nature 515, 274–278. 10.1038/nature13800 25307057 PMC4366007

[B6] Dimidschstein J. Chen Q. Tremblay R. Rogers S. L. Saldi G. A. Guo L.. (2016). A viral strategy for targeting and manipulating interneurons across vertebrate species. Nat. Neurosci. 19, 1743–1749. 10.1038/nn.4430 27798629 PMC5348112

[B7] Duong T. T. Lim J. Vasireddy V. Papp T. Nguyen H. Leo L.. (2019). Comparative AAV-eGFP transgene expression using vector serotypes 1-9, 7m8, and 8b in human pluripotent stem cells, RPEs, and human and rat cortical neurons. Stem Cells Int. 2019:7281912. 10.1155/2019/7281912 30800164 PMC6360060

[B8] El-Husseini A. E. Craven S. E. Chetkovich D. M. Firestein B. L. Schnell E. Aoki C.. (2000a). Dual palmitoylation of PSD-95 mediates its vesiculotubular sorting, postsynaptic targeting, and ion channel clustering. J. Cell Biol. 148, 159–172. 10.1083/jcb.148.1.159 10629226 PMC2156213

[B9] El-Husseini A. E. Schnell E. Chetkovich D. M. Nicoll R. A. Bredt D. S. (2000b). PSD-95 involvement in maturation of excitatory synapses. Science 290, 1364–1368. 10.1126/science.290.5495.1364 11082065

[B10] Fortin D. A. Tillo S. E. Yang G. Rah J. C. Melander J. B. Bai S.. (2014). Live imaging of endogenous PSD-95 using ENABLED: a conditional strategy to fluorescently label endogenous proteins. J. Neurosci. 34, 16698–16712. 10.1523/JNEUROSCI.3888-14.2014 25505322 PMC4261096

[B11] Gross G. G. Junge J. A. Mora R. J. Kwon H. B. Olson C. A. Takahashi T. T.. (2013). Recombinant probes for visualizing endogenous synaptic proteins in living neurons. Neuron 78, 971–985. 10.1016/j.neuron.2013.04.017 23791193 PMC3779638

[B12] Guang S. Pang N. Deng X. Yang L. He F. Wu L.. (2018). Synaptopathology involved in autism spectrum disorder. Front. Cell. Neurosci. 12:470. 10.3389/fncel.2018.00470 30627085 PMC6309163

[B13] Hargus N. J. Thayer S. A. (2013). Human immunodeficiency virus-1 Tat protein increases the number of inhibitory synapses between hippocampal neurons in culture. J. Neurosci. 33, 17908–17920. 10.1523/JNEUROSCI.1312-13.2013 24198379 PMC3818559

[B14] Huang X. F. Song X. (2019). Effects of antipsychotic drugs on neurites relevant to schizophrenia treatment. Med. Res. Rev. 39, 386–403. 10.1002/med.21512 29785841

[B15] Jorfi M. D’Avanzo C. Tanzi R. E. Kim D. Y. Irimia D. (2018). Human neurospheroid arrays for *in vitro* studies of Alzheimer’s disease. Sci. Rep. 8:2450. 10.1038/s41598-018-20436-8 29402979 PMC5799361

[B17] Kim H. J. Martemyanov K. A. Thayer S. A. (2008a). Human immunodeficiency virus protein Tat induces synapse loss *via* a reversible process that is distinct from cell death. J. Neurosci. 28, 12604–12613. 10.1523/JNEUROSCI.2958-08.2008 19036954 PMC2678679

[B19] Kim H. J. Waataja J. J. Thayer S. A. (2008b). Cannabinoids inhibit network-driven synapse loss between hippocampal neurons in culture. J. Pharmacol. Exp. Ther. 325, 850–858. 10.1124/jpet.107.131607 18310474 PMC2398764

[B18] Kim H. J. Shin A. H. Thayer S. A. (2011). Activation of cannabinoid type 2 receptors inhibits HIV-1 envelope glycoprotein gp120-induced synapse loss. Mol. Pharmacol. 80, 357–366. 10.1124/mol.111.071647 21670103 PMC3164336

[B16] Kim H. J. Thayer S. A. (2009). Lithium increases synapse formation between hippocampal neurons by depleting phosphoinositides. Mol. Pharmacol. 75, 1021–1030. 10.1124/mol.108.052357 19188338 PMC2672813

[B20] Koffie R. M. Hyman B. T. Spires-Jones T. L. (2011). Alzheimer’s disease: synapses gone cold. Mol. Neurodegener. 6:63. 10.1186/1750-1326-6-63 21871088 PMC3178498

[B21] Kopec C. D. Li B. Wei W. Boehm J. Malinow R. (2006). Glutamate receptor exocytosis and spine enlargement during chemically induced long-term potentiation. J. Neurosci. 26, 2000–2009. 10.1523/JNEUROSCI.3918-05.2006 16481433 PMC6674938

[B22] Lau A. C. Cui H. Tymianski M. (2007). The use of propidium iodide to assess excitotoxic neuronal death in primary mixed cortical cultures. Methods Mol. Biol. 399, 15–29. 10.1007/978-1-59745-504-6_2 18309922

[B23] Lee J. H. Durand R. Gradinaru V. Zhang F. Goshen I. Kim D. S.. (2010). Global and local fMRI signals driven by neurons defined optogenetically by type and wiring. Nature 465, 788–792. 10.1038/nature09108 20473285 PMC3177305

[B24] Lin L. Lo L. H.-Y. Lyu Q. Lai K.-O. (2017). Determination of dendritic spine morphology by the striatin scaffold protein STRN4 through interaction with the phosphatase PP2A. J. Biol. Chem. 292, 9451–9464. 10.1074/jbc.m116.772442 28442576 PMC5465475

[B25] Liu R.-J. Lee F. S. Li X.-Y. Bambico F. Duman R. S. Aghajanian G. K. (2012). Brain-derived neurotrophic factor Val66Met allele impairs basal and ketamine-stimulated synaptogenesis in prefrontal cortex. Biol. Psychiatry 71, 996–1005. 10.1016/j.biopsych.2011.09.030 22036038 PMC3290730

[B26] Lubbert M. Goral R. O. Satterfield R. Putzke T. van den Maagdenberg A. M. Kamasawa N.. (2017). A novel region in the Ca_V_2.1 α_1_ subunit C-terminus regulates fast synaptic vesicle fusion and vesicle docking at the mammalian presynaptic active zone. Elife 6:e28412. 10.7554/eLife.28412 28786379 PMC5548488

[B27] Luine V. (2016). Estradiol: mediator of memories, spine density and cognitive resilience to stress in female rodents. J. Steroid Biochem. Mol. Biol. 160, 189–195. 10.1016/j.jsbmb.2015.07.022 26241030 PMC4734902

[B28] Miller E. C. Zhang L. Dummer B. W. Cariveau D. R. Loh H. Law P. Y.. (2012). Differential modulation of drug-induced structural and functional plasticity of dendritic spines. Mol. Pharmacol. 82, 333–343. 10.1124/mol.112.078162 22596350 PMC3400837

[B29] Mishra A. Kim H. J. Shin A. H. Thayer S. A. (2012). Synapse loss induced by interleukin-1β requires pre- and post-synaptic mechanisms. J. Neuroimmune Pharmacol. 7, 571–578. 10.1007/s11481-012-9342-7 22311599 PMC3415563

[B30] Montesinos M. S. Satterfield R. Young S. M. Jr. (2016). Helper-dependent adenoviral vectors and their use for neuroscience applications. Methods Mol. Biol. 1474, 73–90. 10.1007/978-1-4939-6352-2_5 27515075

[B31] Nieland T. J. Logan D. J. Saulnier J. Lam D. Johnson C. Root D. E.. (2014). High content image analysis identifies novel regulators of synaptogenesis in a high-throughput RNAi screen of primary neurons. PLoS One 9:e91744. 10.1371/journal.pone.0091744 24633176 PMC3954765

[B32] Nishijima H. Suzuki S. Kon T. Funamizu Y. Ueno T. Haga R.. (2014). Morphologic changes of dendritic spines of striatal neurons in the levodopa-induced dyskinesia model. Mov. Disord. 29, 336–343. 10.1002/mds.25826 24573720

[B33] Palmer D. Ng P. (2003). Improved system for helper-dependent adenoviral vector production. Mol. Ther. 8, 846–852. 10.1016/j.ymthe.2003.08.014 14599819

[B34] Pruunsild P. Bengtson C. P. Bading H. (2017). Networks of cultured iPSC-derived neurons reveal the human synaptic activity-regulated adaptive gene program. Cell Rep. 18, 122–135. 10.1016/j.celrep.2016.12.018 28052243 PMC5236011

[B35] Raybuck J. D. Hargus N. J. Thayer S. A. (2017). A GluN2B-selective NMDAR antagonist reverses synapse loss and cognitive impairment produced by the HIV-1 protein tat. J. Neurosci. 37, 7837–7847. 10.1523/JNEUROSCI.0226-17.2017 28716964 PMC5559761

[B36] Saylor D. Dickens A. M. Sacktor N. Haughey N. Slusher B. Pletnikov M.. (2016). HIV-associated neurocognitive disorder—pathogenesis and prospects for treatment. Nat. Rev. Neurol. 12, 234–248. 10.1038/nrneurol.2016.27 26965674 PMC4937456

[B37] Sharma K. Choi S. Y. Zhang Y. Nieland T. J. Long S. Li M.. (2013). High-throughput genetic screen for synaptogenic factors: identification of LRP6 as critical for excitatory synapse development. Cell Rep. 5, 1330–1341. 10.1016/j.celrep.2013.11.008 24316074 PMC3924421

[B38] Shen X. Yeung H. T. Lai K. O. (2019). Application of human-induced pluripotent stem cells (hiPSCs) to study synaptopathy of neurodevelopmental disorders. Dev. Neurobiol. 79, 20–35. 10.1002/dneu.22644 30304570

[B40] Shin A. H. Kim H. J. Thayer S. A. (2012). Subtype selective NMDA receptor antagonists induce recovery of synapses lost following exposure to HIV-1 Tat. Br. J. Pharmacol. 166, 1002–1017. 10.1111/j.1476-5381.2011.01805.x 22142193 PMC3417425

[B39] Shin A. H. Thayer S. A. (2013). Human immunodeficiency virus-1 protein Tat induces excitotoxic loss of presynaptic terminals in hippocampal cultures. Mol. Cell. Neurosci. 54, 22–29. 10.1016/j.mcn.2012.12.005 23267846 PMC3622188

[B41] Sirenko O. Parham F. Dea S. Sodhi N. Biesmans S. Mora-Castilla S.. (2019). Functional and mechanistic neurotoxicity profiling using human iPSC-derived neural 3D cultures. Toxicol. Sci. 167, 58–76. 10.1093/toxsci/kfy218 30169818 PMC6317428

[B42] Smafield T. Pasupuleti V. Sharma K. Huganir R. L. Ye B. Zhou J. (2015). Automatic dendritic length quantification for high throughput screening of mature neurons. Neuroinformatics 13, 443–458. 10.1007/s12021-015-9267-4 25854493 PMC4600005

[B43] Spicer T. P. Hubbs C. Vaissiere T. Collia D. Rojas C. Kilinc M.. (2018). Improved scalability of neuron-based phenotypic screening assays for therapeutic discovery in neuropsychiatric disorders. Mol. Neuropsychiatry 3, 141–150. 10.1159/000481731 29594133 PMC5836166

[B44] Taoufik E. Kouroupi G. Zygogianni O. Matsas R. (2018). Synaptic dysfunction in neurodegenerative and neurodevelopmental diseases: an overview of induced pluripotent stem-cell-based disease models. Open Biol. 8:180138. 10.1098/rsob.180138 30185603 PMC6170506

[B46] Vokes M. S. Carpenter A. E. (2008). Using CellProfiler for automatic identification and measurement of biological objects in images. Curr. Protoc. Mol. Biol. 14:14.17. 10.1002/0471142727.mb1417s82 18425761

[B47] Waataja J. J. Kim H. J. Roloff A. M. Thayer S. A. (2008). Excitotoxic loss of post-synaptic sites is distinct temporally and mechanistically from neuronal death. J. Neurochem. 104, 364–375. 10.1111/j.1471-4159.2007.04973.x 17944868

[B49] Zhang X. Green M. V. Thayer S. A. (2019). HIV gp120-induced neuroinflammation potentiates NMDA receptors to overcome basal suppression of inhibitory synapses by p38 MAPK. J. Neurochem. 148, 499–515. 10.1111/jnc.14640 30520043 PMC6379117

[B48] Zhang X. Thayer S. A. (2018). Monoacylglycerol lipase inhibitor JZL184 prevents HIV-1 gp120-induced synapse loss by altering endocannabinoid signaling. Neuropharmacology 128, 269–281. 10.1016/j.neuropharm.2017.10.023 29061509 PMC5752128

