# Supplementary material for: Automated Live-Cell Imaging of Synapses in Rat and Human Neuronal Cultures
Source: Front Cell Neurosci. 2019 Oct 17;13:467. doi: 10.3389/fncel.2019.00467 (PMC6811609; doi:10.3389/fncel.2019.00467)
Supplement: Supplementary file 1 [file Table_1.DOCX]

| Table 1: Image Analysis Threshold Settings | | | | |
| --- | --- | --- | --- | --- |
| Figure | Green Puncta  T= I_green_+ *x_G_* (SD_green_)  (*x_G_*) | Green Puncta Area  (number of pixels (minimum-maximum) | Red Mask  T = *x_R_* (I_red_)  (*x_R_*) | Red Particle  T = I_red_ + *x_P_* (SD_red_)  (*x_P_*) |
| 2A | 3.5 | 8-80 | 0.5 | 0.25 |
| 2Ci | 1.5 | 8-80 | 0.5 | - |
| 2Cii | 3.5 | 8-80 | 0.5 | - |
| 2Ciii | 5.5 | 8-80 | 0.5 | - |
| 3 | 3.5 | 8-80 | 0.25 | 0.25 |
| 4A | 2.5 | 4-60 | 0.25 | 0.25 |
| 4C | 2.0 | 4-60 | I_red_ + SD_red_ | 0.25 |
| 5A-B | 5.0 | 3-30 | 0.5 | 0.25 |
| Definitions: T, threshold; I_green_, average green MIP intensity; SD_green_ , green MIP standard deviation; I_red_, average red MIP intensity; SD_red_ , red MIP standard deviation; *x_G_* , scaling factor for green puncta counting determined as described in text and Figure 2B; *x_R_*, scaling factor for red mask; *x_P_*, scaling factor for particle analysis. | | | | |
